# Anaerobic consortia and waste treatment: An annotated selection of World Wide Web sites relevant to the topics in *microbial biotechnology*

**DOI:** 10.1111/1751-7915.13311

**Published:** 2018-08-22

**Authors:** Lawrence P. Wackett

**Affiliations:** ^1^ Department of Biochemistry, Molecular Biology and Biophysics BioTechnology Institute University of Minnesota St. Paul MN 55108 USA

## Synthetic consortia for wastewater

https://www.frontiersin.org/articles/10.3389/fenvs.2016.00030/full

This study investigated bioaugmentation of consortia that function in terms of enhancing chemical oxygen demand decrease in wastewater treatments.

## Structure of microbial consortia in activated sludge

https://www.nature.com/articles/s41598-017-17743-x

This study focused on defining microbial populations involved in chemical industrial wastewater treatments. Major genera identified were *Thauera, Macellibacteroides, Desulfomicrobium* and *Thiobacillus*.

## Microbial consortia for dairy wastewater treatment

https://link.springer.com/article/10.1007/s12257-013-0517-8

Diary wastewater is rich in fat and proteins and so is ideally composed of different members than a chemical or municipal water treatment system. Major genera of bacteria are *Bacillus, Serratia, Lactococcus, Enterococcus, Stenotrophomonas, Klebsiella* and *Escherichia*.

## Anaerobic consortia converting tetramethylammonium to methane

https://www.hindawi.com/journals/archaea/2017/2170535/

This study examined a highly specific microbial consortia using a 16S rRNA gene sequencing and proteomics approach.

## Recycling anaerobic wastewater treatment system

https://patents.google.com/patent/US7520990

This patent describes an anaerobic wastewater treatment with a recycling system designed to recapture anaerobic microbial consortia particles escaping from the bioreactor tank.

## Trace metals and methanogenic consortia

http://edepot.wur.nl/40320

This Ph.D. thesis describes studies on effects of metals on anaerobic methanogenic consortia.

## Anaerobic digestion: Climatetech wiki

http://www.climatetechwiki.org/technology/jiqweb-anbt

This webpage offers a useful primer on anaerobic digestion processes and systems.

## Wastewater treatment glossary

http://www.onsiteconsortium.org/Glossary2009.pdf

This site provides a useful guide for common and specialized terminology relevant to wastewater treatment and anaerobic consortia.

## Microbiomes and resource management

https://www.omicsonline.org/open-access/a-new-perspective-on-microbiome-and-resource-management-in-wastewater-systems-2155-952X-1000184.php?aid=54389

This review provides a good overview of microbial wastewater treatment and describes how microbiome research is beginning to impact the field.

## Microbe detectives

https://microbedetectives.com/wastewater-treatment/

This company website describes their service of analyzing water systems for their microbial contents, with the goal of aiding in process optimization.

## Global water microbiome consortium

http://gwmc.ou.edu

The overall goal of the global water microbiome consortium is to provide insights into the diversity and stability of wastewater microbiomes through a global collaboration with different groups working on these microbiomes.

## Municipal wastewater standards

https://www.epa.gov/npdes/municipal-wastewater

This page put out by the United States Environmental Protection Agency contains numerous useful documents for information and standards.

## Microbiome water summit

http://microbiomewatersummit.com/wastewater-microbiome/

The microbiome water summit is one of many conferences on this emerging theme emphasizing the importance of microbiome composition of water treatment processes.

## Chain elongation of anaerobic consortia

https://pubs.acs.org/doi/abs/10.1021/acs.est.5b04847

The goal of this study was to drive chain elongation of fatty acids with ethanol as the electron donor. The overall goal is to convert organic waste into process chemicals.

